# Sequential Bromelain-Based Enzymatic Debridement in a Near-Total (97%) TBSA Burn: A Case Report

**DOI:** 10.3390/ebj7030046

**Published:** 2026-09-07

**Authors:** Jannik Hinzmann, Sonja Verena Schmidt, Elisabete Macedo Santos, Maria Fueth, Marius Drysch, Yonca Steubing, Christoph Wallner

**Affiliations:** 1 Department of Plastic Surgery, BG University Hospital Bergmannsheil Bochum, Ruhr-University Bochum, 44789 Bochum, Germany; 2 Department of Anesthesiology, BG University Hospital Bergmannsheil Bochum, Ruhr-University Bochum, 44789 Bochum, Germany

**Keywords:** bromelain-based enzymatic debridement, NexoBrid, extensive burns, total body surface area, off-label use

## Abstract

**Highlights:**

**What are the main findings?**

- Sequential bedside enzymatic debridement with NexoBrid enabled complete eschar removal from a 97% TBSA burn within 64 h in five treatment sessions.
- The procedure was completed without transfer to the operating room, major procedure-related bleeding, or transfusion during the enzymatic debridement period.

**What are the implications of the main findings?**

- Sequential enzymatic debridement may offer an alternative to extensive surgical excision in carefully selected patients with very large burns.
- Large-area off-label enzymatic debridement requires specialized expertise, standardized procedural planning, and continuous intensive care monitoring.

**Abstract:**

**Introduction:** Early debridement of burn eschar is a cornerstone of care for patients with extensive burns, yet early large-area surgical excision may be limited by blood loss, physiological instability, and substantial logistical demands. Evidence on bromelain-based enzymatic debridement involving treatment areas greater than 15% total body surface area (TBSA) remains scarce. **Case Report:** A 38-year-old man sustained partial- and full-thickness burns involving 97% TBSA, together with inhalation injury. Sequential enzymatic debridement with NexoBrid was performed at the bedside in five 4-h sessions under intensive care conditions, with 14–27% TBSA treated per session. Complete eschar removal was achieved within 64 h of admission without transfer to the operating room, major procedure-related bleeding, or transfusion during the debridement period. The subsequent course was complicated by sepsis and severe respiratory failure requiring veno-venous extracorporeal membrane oxygenation. Following intensive care treatment, the patient stabilized markedly, ECMO support was discontinued, and near-complete wound coverage was achieved. He died on hospital day 87 following acute intrapulmonary and endotracheal hemorrhage of unclear etiology. **Conclusions:** This case illustrates the technical feasibility of early, sequential bedside enzymatic debridement in a near-total burn and may inform its carefully selected off-label use in highly specialized burn centers.

## 1. Introduction

Despite advances in resuscitation strategies, intensive care protocols, and surgical burn management, severe burn injuries involving extensive total body surface area (TBSA) still lead to substantial morbidity and mortality, primarily due to profound systemic inflammation and subsequent multiple organ dysfunction syndrome (MODS) [1,2,3,4]. Therefore, early removal of necrotic tissue has become a central component of modern burn care. Although definitions of early burn wound excision vary across the literature [5,6,7,8], the term “early” is used throughout this report to refer to debridement performed within the first 72 h after injury. Despite the possible advantage of mitigating burn shock, early surgical debridement in burn patients remains a highly invasive procedure and may be limited by blood loss, physiologic instability, operating room logistics, and the need for substantial personnel and infrastructural resources.

Since its approval by the European Medicines Agency (EMA) in 2012, bromelain-based enzymatic debridement with NexoBrid^®^ has emerged as a more selective alternative to surgical eschar removal which can be performed at the bedside and may reduce blood loss and preserve viable dermis in partial-thickness burns [9,10,11]. However, the approved treatment area is limited to a maximum of 15% TBSA. Accordingly, application of NexoBrid to more than 15% TBSA constitutes off-label use [12]. Consequently, clinical evidence regarding the use of NexoBrid in extensive burns remains limited, but initial studies suggest that off-label use may be feasible and potentially safe [13].

This report describes the technical and physiological course of sequential enzymatic debridement in a patient with near-total burn injury involving 97% TBSA. Four of the five treatment sessions individually involved treatment areas of more than 15% TBSA, and complete enzymatic debridement of all 97% TBSA was achieved within the first 64 h.

## 2. Case Presentation

**Figure 1:**
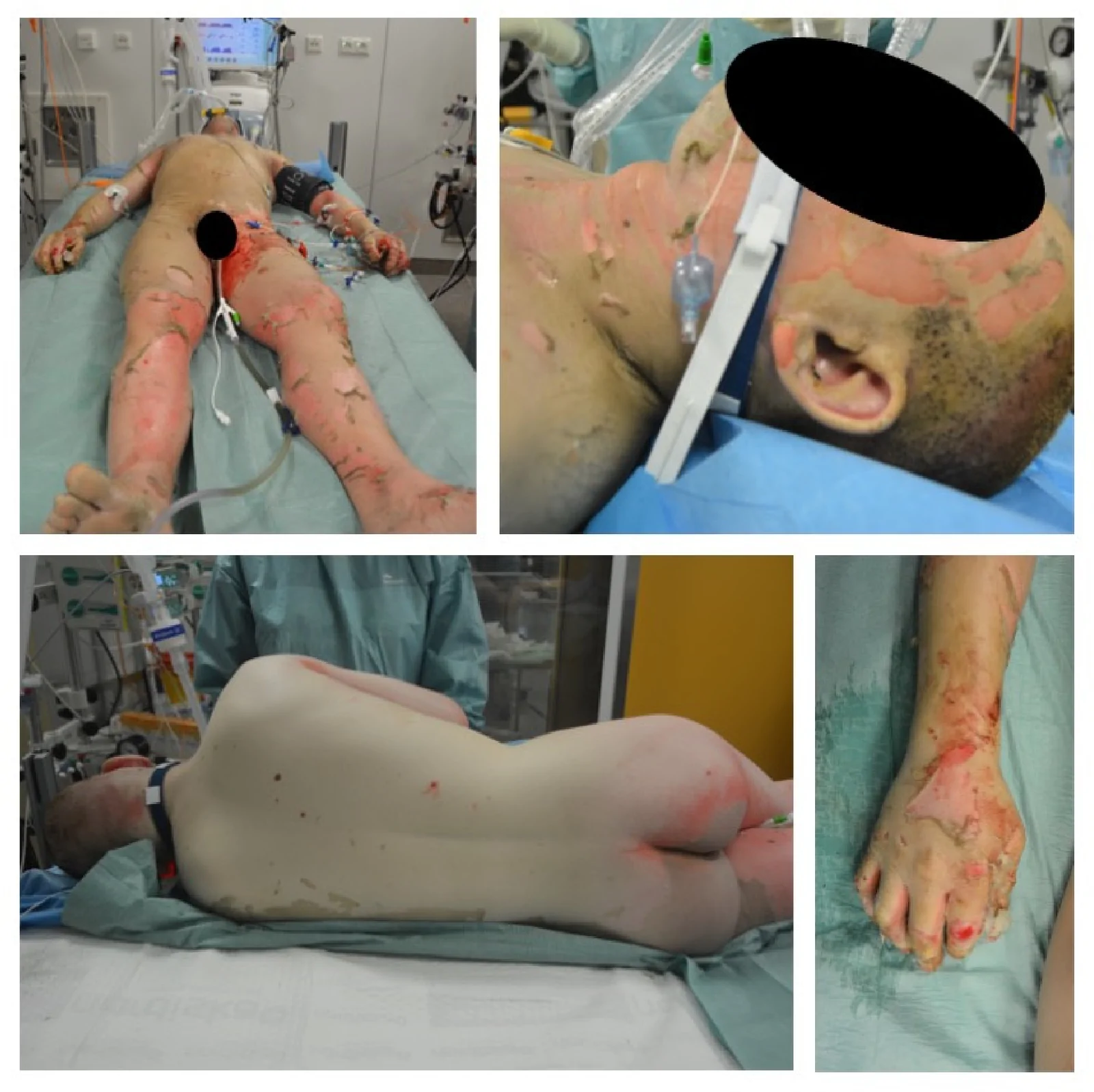
Photographic documentation after initial treatment and stabilization of the patient in the ICU, demonstrating partial- and full-thickness burns involving 97% TBSA.

A 38-year-old man was admitted to our burn center approximately 1 h after injury with partial- and full-thickness burns involving 97% TBSA and inhalation injury (Figure 1). The day of admission was defined as hospital day 1. The injury resulted from an explosion of a gas cylinder at home, leading to complete destruction of his apartment. Upon arrival of emergency medical services, the patient was still conscious and ambulatory. Because of the severity of the injury, endotracheal intubation was performed immediately, and the patient was transferred directly to the burn center.

Initial clinical examination, including whole-body CT, excluded additional traumatic injuries. The patient was transferred to the intensive care burn unit for further treatment. Reassessment confirmed 97% TBSA involvement, comprising 15% superficial-partial-thickness burns, 67% deep-partial-thickness burns and 15% full-thickness burns. Only the soles of the feet and small areas of the face, scrotum, and lower legs remained unaffected. Burn depth was determined clinically by experienced burn surgeons, without laser Doppler imaging, histological confirmation, or other objective assessment techniques. The patient presented no comorbidities. Abbreviated Burn Severity Index (ABSI) score was 14, equivalent to an estimated survival probability of less than 10% [14].

Under initial intensive care treatment, including aggressive fluid resuscitation and dual catecholamine support, the patient remained sufficiently stable. Given the predominance of partial-thickness burns and the aim of achieving complete debridement as early as possible, sequential enzymatic debridement using NexoBrid was selected as the primary debridement strategy.

Large-area enzymatic debridement was prepared with operative-level procedural readiness, including a defined sequence of debridement steps and a strategy for temporary and permanent coverage. Before each application, hemodynamic, respiratory, and metabolic stability were reassessed. Extended laboratory testing was obtained, including blood count, electrolytes, coagulation parameters, ROTEM analysis, and factor XIII. Packed red blood cells (PRBCs) were cross-matched and kept immediately available, generally at a ratio of approximately one unit per 10% TBSA scheduled for treatment. Initial wound bed preparation included cleansing and removal of loose devitalized epidermis. The first NexoBrid application was initiated without dedicated presoaking. Thereafter, all wounds were covered with polyhexanide-containing dressings and paraffin gauze, with routine dressing changes once daily. No additional dedicated presoaking was performed immediately before subsequent NexoBrid applications. Also, no routine systemic antibiotic prophylaxis was administered solely for enzymatic debridement. Throughout the debridement period, the patient remained intubated and mechanically ventilated under continuous analgesia and sedation with sufentanil combined initially with propofol and subsequently with isoflurane. In anticipation of increased procedural stimulation and pain, particularly during the first 30–60 min after NexoBrid application, sedation was temporarily deepened to a Richmond Agitation-Sedation Scale (RASS) score of −5. Thereafter, analgesia and sedation were gradually reduced and titrated according to the ongoing procedural requirements and the patient’s clinical condition.

Application of NexoBrid required three to four team members for approximately 30–45 min whereas subsequent removal required at least two team members. During application, one physician was dedicated to airway, ventilation, monitoring, and continuous infusion therapy. After application, one team member remained near the patient for at least 30 min to detect early hemodynamic instability, bleeding, or dressing-related problems. Thereafter, the patient remained under continuous intensive care unit (ICU) monitoring. Treatment was sequenced from the head and extremities to the trunk because these areas were technically more accessible and allowed additional preparation time before treating the trunk. Blood gas analysis was intensified from the standard 4–6-h ICU interval to 2-h intervals during enzymatic debridement. Based on institutional experience with large-area NexoBrid applications, the crystalloid infusion rate was increased by approximately 50% before each treatment to anticipate the early hemodynamic response typically observed during the first 30–60 min after application. Thereafter, fluid administration was reduced and further adjusted according to the patient’s overall clinical and hemodynamic status, with additional crystalloid boluses available if required.

**Figure 2:**
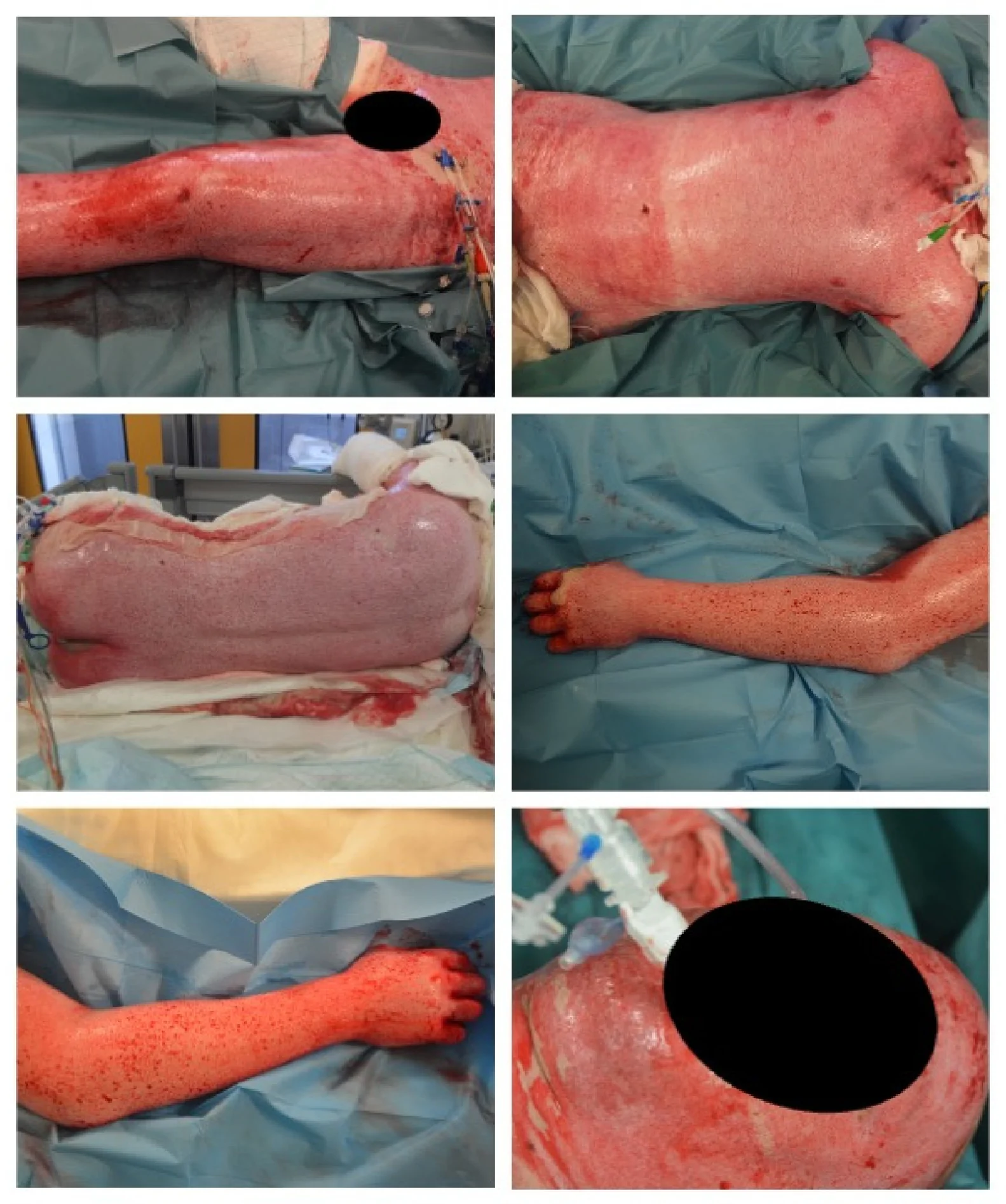
Photographic documentation of the wound bed immediately after enzymatic debridement and cleansing. Most areas demonstrate a predominantly uniform white wound bed with major focal hemorrhages, consistent with deep partial-thickness burns.

Enzymatic debridement was initiated 3.5 h after ICU admission, with each application lasting 4 h. Treatment began with the head and both upper extremities at hospital hour 3.5, covering 27% TBSA, followed by the right lower extremity at hour 19.5, covering 17% TBSA. On hospital day 2, the left lower extremity and lower abdomen were treated at hour 33, covering 21% TBSA. On hospital day 3, debridement continued with the upper abdomen and thorax at hour 48, covering 14% TBSA, and was completed with the back at hour 60, covering the remaining 18% TBSA. Each anatomical region was treated once, and NexoBrid was not reapplied to previously treated wound areas. The mean treated area per session was 19.4% TBSA. Complete enzymatic eschar removal was achieved within 64 h after admission without transport to the operating room (Figure 2).

After each application, the agent was removed, the wound bed was cleansed and standardized photographic documentation was obtained. Completeness of enzymatic debridement was assessed clinically by visual inspection, based on the absence of residual adherent eschar and the appearance of the exposed wound bed. Polyhexanide-based dressings and paraffin gauze were reapplied and inspected for bleeding 2 h later. Thereafter these dressings were changed daily until the respective wound area underwent coverage. No additional surgical eschar debridement was required after NexoBrid removal. During subsequent wound-coverage procedures, the wound bed was only refreshed as part of routine preparation for coverage. Recovery intervals of at least 8 h were scheduled between sessions and guided by hemodynamic stability, respiratory status, temperature control, and metabolic recovery.

**Figure 3:**
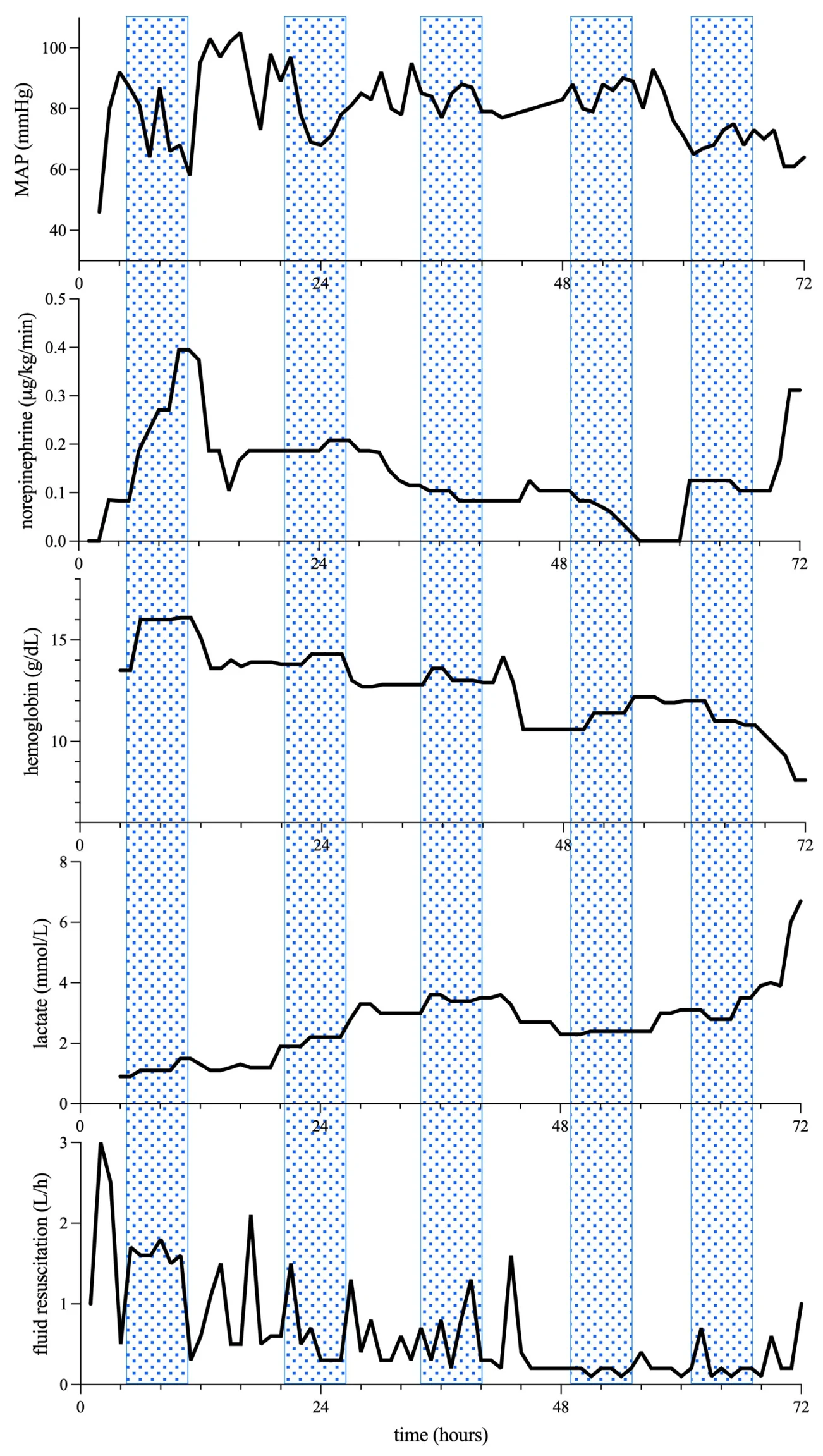
Hemodynamic and metabolic parameters during the first 71 h after admission. Mean arterial pressure (MAP), norepinephrine dose, hemoglobin concentration, serum lactate levels, and fluid resuscitation are shown over time. Blue shaded bars indicate the respective 4-h periods of sequential enzymatic debridement. Time zero corresponds to hospital day 1, 00:00; ICU admission occurred at 01:00.

During the first 24 h, the patient received 26 L of crystalloid fluid and 1000 mL of 20% albumin solution. During the subsequent 24 h, an additional 12 L of crystalloid fluid and 300 mL of 20% albumin solution were administered. In comparison, the Parkland-Baxter formula predicted approximately 31 L of crystalloid resuscitation during the first 24 h. High-dose vitamin C was administered during the first 24 h to reduce capillary leak. Catecholamine support could be gradually reduced while maintaining stable circulation. Hemoglobin levels declined from 13.5 g/dL on admission to 8.5 g/dL by the end of hospital day 3, without the need for PRBCs during the enzymatic debridement period (Figure 3). No major bleeding event occurred during or immediately after the debridement sessions.

Despite adequate fluid resuscitation, serum lactate levels increased and pulmonary function deteriorated, requiring escalation of ventilatory support. On the evening of hospital day 3, approximately 5 h after completion of the final enzymatic debridement session, veno-venous extracorporeal membrane oxygenation (V-V ECMO) was initiated. Continuous veno-venous hemofiltration (CVVH) was commenced on hospital day 4. In this context, transfusion of two units of PRBCs became necessary for the first time. At the transition from hospital day 3 to 4, sepsis with *Streptococcus pneumoniae* bacteremia was diagnosed. Under intensified intensive care treatment, the patient stabilized again. V-V ECMO support was maintained for 13 days and was successfully discontinued on hospital day 16.

Due to the absence of sufficient donor sites for immediate autologous skin grafting, temporary wound coverage was achieved using allogeneic skin grafts on hospital days 6 and 13. The hands were temporarily covered with fish skin grafts. On hospital day 34, autologous split-thickness skin grafting (mesh and MEEK technique) was performed for the hands and forearms, using grafts harvested from regenerated skin of the scalp and scrotum. On hospital day 5, skin biopsies were obtained for cultivation of keratinocyte sheets at an external laboratory, which were transplanted on hospital day 44 to both upper and lower extremities and the anterior torso.

Owing to sufficient residual dermal stem cell populations, spontaneous epithelialization occurred partly on the anterior torso and predominantly on the back. Residual defects, predominantly consisting of hypergranulation tissue and involving approximately 9% TBSA of the back, were treated on hospital day 83 using an autologous cell spray-on suspension and a polylactide-based synthetic skin substitute. Following this intervention, wound coverage was nearly complete. The final wound assessment on hospital day 86 showed that, apart from the recently treated approximately 9% TBSA on the back, only small additional residual wound areas involving approximately 5% TBSA remained.

**Figure 4:**
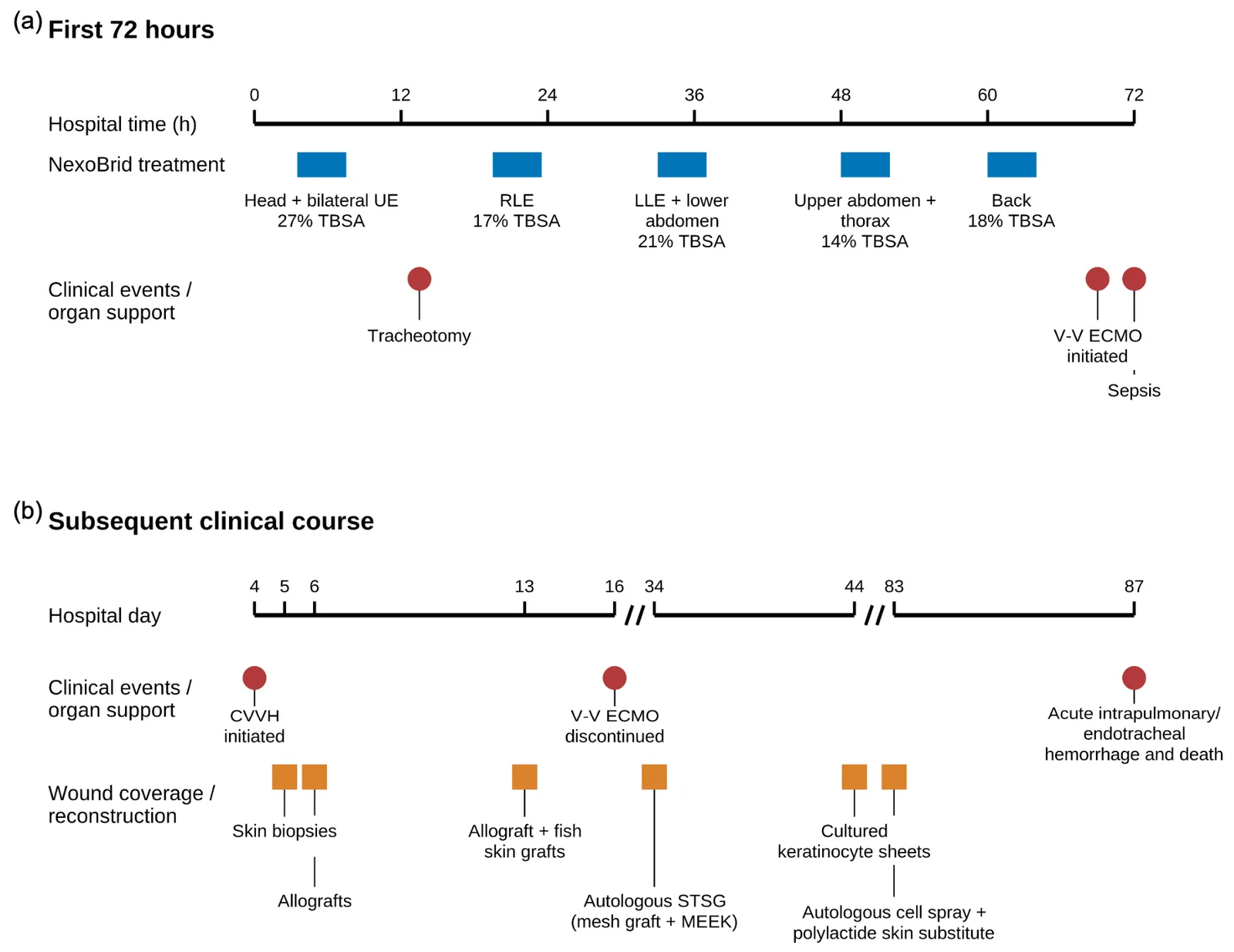
Timeline of sequential enzymatic debridement, major clinical events, and subsequent wound coverage. (**a**) First 72 h after admission, showing the timing and extent of the five sequential NexoBrid applications and major clinical events. (**b**) Subsequent clinical course, including organ support and wound-coverage procedures, displayed using a discontinuous time axis. Blue bars indicate NexoBrid treatment sessions, red circles indicate major clinical events or organ-support interventions, and orange squares indicate wound-coverage procedures. TBSA, total body surface area; UE, upper extremities; RLE, right lower extremity; LLE, left lower extremity; V-V ECMO, veno-venous extracorporeal membrane oxygenation; CVVH, continuous veno-venous hemofiltration; STSG, split-thickness skin graft; MEEK, modified Meek micrografting technique.

At the general clinical assessment on the same day, the patient was awake, responsive, fully oriented, regularly using a speaking valve, and able to sit at the edge of the bed. During the clinical course, the patient developed multiple episodes of pneumonia, including pulmonary aspergillosis requiring antibiotic and antimycotic therapy as well as repeated bronchoscopic interventions. Due to copious bronchial secretions, frequent endotracheal suctioning was required. On hospital day 87, without preceding intervention, the patient acutely developed intrapulmonary and endotracheal hemorrhage with severe hypoxia and hemorrhagic shock. Despite immediate and extensive resuscitative measures, adequate oxygenation could not be re-established, and the patient died. Since an autopsy was not performed, the etiology of the hemorrhage could not be conclusively determined. The overall treatment course, including the sequential enzymatic debridement sessions, major clinical events, and subsequent wound coverage, is summarized in Figure 4.

## 3. Discussion

For more than a decade, enzymatic debridement using NexoBrid has been established as an effective method for selective eschar removal in partial- and full-thickness burns and represents a validated alternative to surgical excision [15,16]. However, approved use remains limited to burn areas of up to 15% TBSA [12]. In the present case, complete sequential enzymatic debridement was technically achieved in a patient with near-total TBSA involvement (97%), representing an extent far beyond the currently approved indication.

Evidence on individual NexoBrid applications involving more than 15% TBSA remains scarce [13,17]. Evidence on repeated sequential applications resulting in substantially larger cumulative treated areas is even more limited. Krauss et al. reported a case in which NexoBrid was applied in three off-label sessions covering a total of 54% TBSA. In that case, enzymatic debridement was delayed until hospital day five, and the patient died shortly after the third session on day 8 due to acute lung and heart failure [18]. In a retrospective review by Bowers et al., one patient underwent enzymatic debridement in three sessions of approximately 20% TBSA each, covering a total of about 60% TBSA. Although this patient reportedly survived, detailed information regarding the clinical course was not provided [19]. To our knowledge, these reports represent the most extensive published cumulative applications of NexoBrid to date, yet they remain significantly below the 97% TBSA treated in the present case.

Burn injuries involving more than 80–90% TBSA are rare and associated with extremely high mortality. A cornerstone of modern burn care is the concept of early excision, which has been shown to reduce systemic inflammation, infection rates, and mortality [5,7,8]. However, the implementation of early surgical excision requires substantial logistical resources and may be limited by patient instability, bleeding risk, or restricted availability of operating room capacity. The present case demonstrates that, as an alternative, complete enzymatic eschar removal can be technically achieved at the bedside within this early time frame. Sequential treatment allowed repeated assessment of the patient’s physiological response to each session, provided opportunities for intensive medical stabilization between treatments, and enabled additional necessary interventions, such as tracheotomy, without delaying wound management. Although the protocol remained a highly resource-intensive ICU procedure requiring meticulous planning and experienced staff, it avoided transfer to the operating room and extensive operative excision during the initial debridement period. By dividing treatment into sequential bedside sessions, the approach may also allow procedural resource demands to be distributed over time and planned more flexibly rather than concentrated around major operative procedures.

Despite the poor prognosis associated with the extent of injury and an extremely high ABSI score, the patient survived the entire phase of wound debridement as well as the subsequent definitive coverage of nearly all remaining burn areas. Nevertheless, severe systemic deterioration occurred during the early clinical course and required extracorporeal organ support. In the setting of a near-total burn injury, the requirement for V-V ECMO and CVVH should be interpreted in the context of the profound systemic injury and does not, by itself, indicate failure of the local debridement strategy. Furthermore, enzymatic debridement selectively preserves viable dermal structures in partial-thickness burns, which may reduce the overall need for autologous skin grafting and promote spontaneous epithelialization, as observed in parts of the present case.

The early development of sepsis represents an important limitation when interpreting procedural safety. No routine systemic antibiotic prophylaxis was administered solely for enzymatic debridement, consistent with current guidelines, which do not recommend routine systemic antibiotic prophylaxis in burn patients [20,21]. Given the profound infectious risk associated with near-total burn injury and inhalation injury, early bloodstream infection may plausibly occur irrespective of the debridement modality. Nevertheless, a contributory role of extensive enzymatic debridement cannot be excluded.

The decision to apply enzymatic debridement requires careful patient selection and consideration of multiple clinical factors, including burn depth, TBSA involvement, associated traumatic injuries, comorbidities, and institutional experience. Accordingly, this case should not be interpreted as definitive evidence of safety in near-total burns, but as a technical and physiological report that may inform carefully selected off-label use in highly specialized burn centers.

## 4. Conclusions

This fatal case demonstrates that sequential bedside enzymatic debridement of near-total TBSA burns can be technically achieved within the defined early time frame under intensive care conditions, enabling complete eschar removal. Although this approach cannot prevent the systemic complications inherent to extreme burn injury, it may represent a more flexible alternative to extensive surgical excision in carefully selected patients, provided that substantial expertise, meticulous planning, and continuous intensive care monitoring are available.

## Data Availability

All relevant data are included in the article. Additional clinical data are not publicly available due to patient confidentiality.
